# A Rare Case of Morpheaform Basal Cell Carcinoma With Lacrimal Sac Invasion

**DOI:** 10.1155/cris/7294289

**Published:** 2026-04-02

**Authors:** Alper Geyik, Ahmet Özdemir, Tahir Babahan, Fatih Berk Ateşşahin, Banu Lebe, Şeyma Kılıçarslan Özdemir

**Affiliations:** ^1^ Department of Plastic, Reconstructive and Aesthetic Surgery, Dokuz Eylul University, Izmir, Türkiye, deu.edu.tr; ^2^ Department of Pathology, Dokuz Eylul University, Izmir, Türkiye, deu.edu.tr; ^3^ Department of Radiology, Katip Çelebi University, Izmir, Türkiye, ikc.edu.tr

**Keywords:** basal cell carcinoma, lacrimal sac invasion, morpheaform BCC, periorbital tumor

## Abstract

Basal cell carcinoma (BCC) is the most common type of skin cancer and is a significant focus in plastic surgery practice. BCC also represents the most common epithelial tumor of the periocular region, particularly affecting the medial canthus and lower eyelid. Multiple studies have suggested that tumor recurrence is more likely when BCC arises in specific anatomical sites, such as the medial canthus, or involves aggressive histological subtypes like morpheaform or sclerosing variants. BCC located near the medial canthus may extend to involve the lacrimal drainage system and adjacent ocular structures if not treated promptly. Nevertheless, direct invasion of the lacrimal canaliculus or sac remains uncommon, with only a few cases reported in the literature to date. In this report, we present a case of morpheaform BCC with invasion of the lacrimal sac and discuss the surgical management and reconstructive approach.

## 1. Introduction

Basal cell carcinoma (BCC) is the most common type of non‐melanoma skin cancer worldwide. More than 75% of BCC cases occur in the head and neck region, with approximately 20% in the periocular area [1]. BCC is generally slow‐growing and rarely metastasizes; the morpheaform (infiltrative) subtype can result in local destruction and frequently leads to recurrence. Recent research indicates that the risk of incomplete excision is three times higher in morpheaform BCC (12.6%) compared to nodular BCC (4%) [2].

Due to the complex anatomy of the periocular region and its significant functional and esthetic roles, early treatment of these tumors is crucial. Unfortunately, patients sometimes do not present at an early stage, or specific subtypes of these tumors progress rapidly, resulting in functional impairments, loss of aesthetic structures, or even vision‐threatening complications, particularly in the periorbital area. Increased recurrence rates have been associated with certain anatomical sites, notably the medial canthus, as well as particular histological subtypes like morpheaform or sclerosing BCC [1]. For aggressive BCC subtypes, such as morpheaform, treatment planning should be carried out by surgeons experienced in the region’s anatomy and possessing the necessary reconstructive skills.

The primary treatment for periocular BCC is radical surgical excision, often employing the Mohs micrographic technique. Achieving optimal functional and aesthetic outcomes following complete tumor excision and reconstruction is essential. In the presence of orbital invasion by high‐risk aggressive BCC, radical exenteration should be considered to prevent further extension and recurrence [3].

In this report, we present a rare case of morpheaform BCC invading the lacrimal sac and describe the surgical management and reconstructive strategy used in this patient.

## 2. Case Presentation

**Figure 1:**
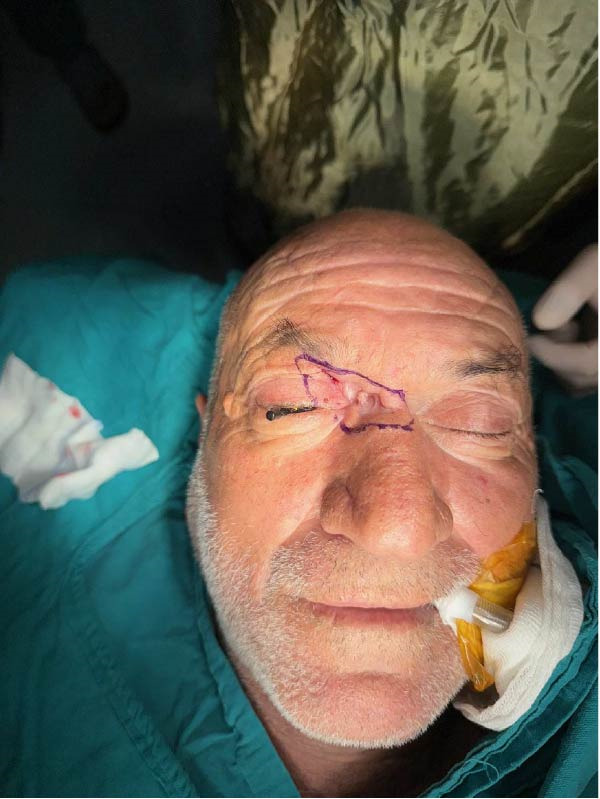
Preoperative image of patient.

A 62‐year‐old man with no history of immunosuppressive drug use or chronic disease presented to the ophthalmology department of another healthcare institution with a progressively enlarging ulcerative lesion in the right medial canthal region, which had developed over a 6‐month period. Upon evaluation, a diagnostic excisional biopsy was performed, which demonstrated persistent morpheaform BCC at the basal margin. The patient was subsequently referred to a plastic surgery service for wide local excision; however, he did not attend the consultation for approximately 7 months due to social circumstances. During this interval, the lesion continued to enlarge, and the patient did not receive any treatment. Upon admission, a depressed, scar‐like mass measuring 5 cm × 2 cm was observed extending from the medial canthal region to the mid‐nasal radix and supraorbital rim, involving both the upper and lower eyelids (Figure 1).

**Figure 2:**
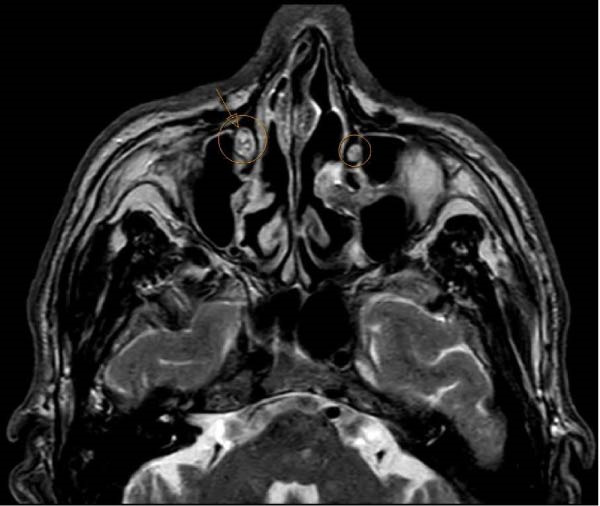
Preoperative MRI, lacrimal sac invasion.

Palpation suggested possible subcutaneous extension, particularly superiorly in the upper eyelid, reaching beneath the lateral eyebrow. Epiphora and conjunctivitis were also noted. A preoperative magnetic resonance imaging (MRI) was performed to evaluate the lacrimal system and globe. MRI revealed a lesion in the right medial canthus extending to the upper and lower eyelids, nasolacrimal duct, and nasal root but not to the post‐septal area (Figure 2).

No involvement of the globe or conjunctiva was detected on MRI, which was consistent with the ophthalmologic examination. Consequently, wide excision with frozen section analysis was planned to achieve negative tumor margins without exenteration.

**Figure 3:**
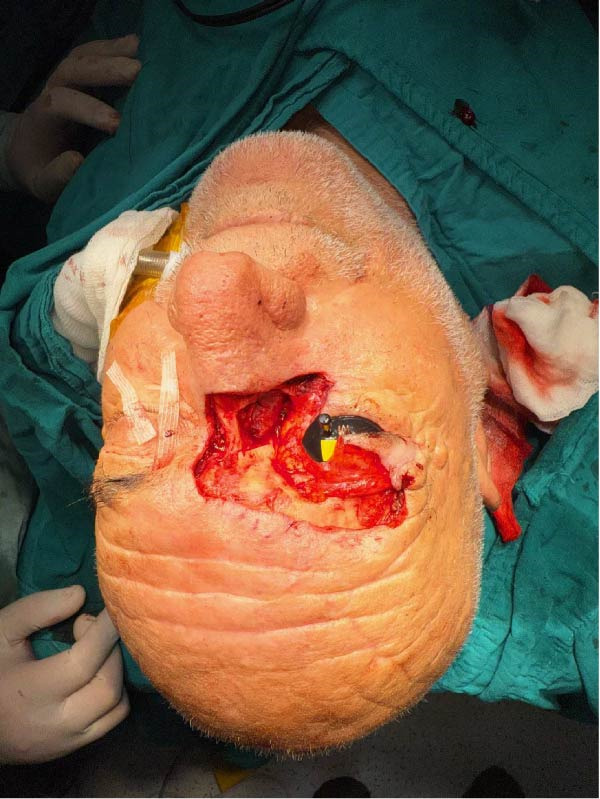
After negative frozen pathology result, complete resection of tumor.

**Figure 4:**
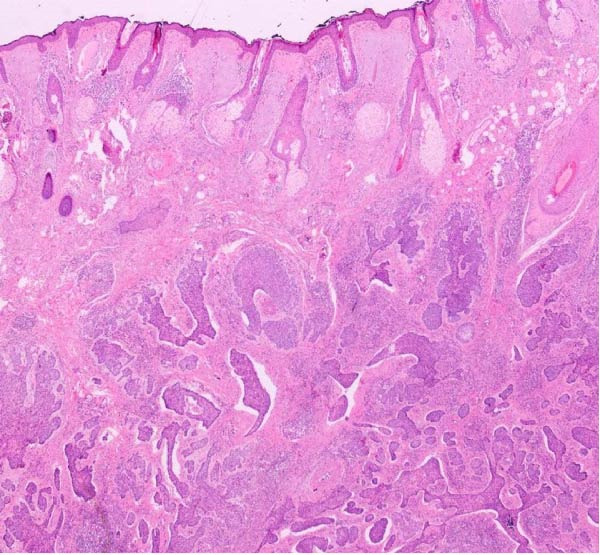
Histopathological photograph infiltrative basal cell carcinoma.

**Figure 5:**
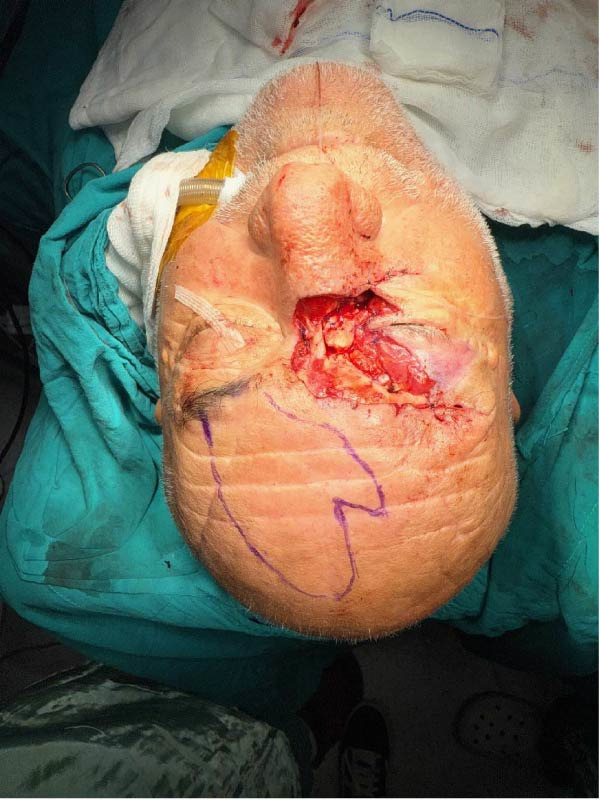
Planning of the forehead flap. The defect is partially reduced somewhat inferiorly and laterally by advancement and suturing before flap elevation.

**Figure 6:**
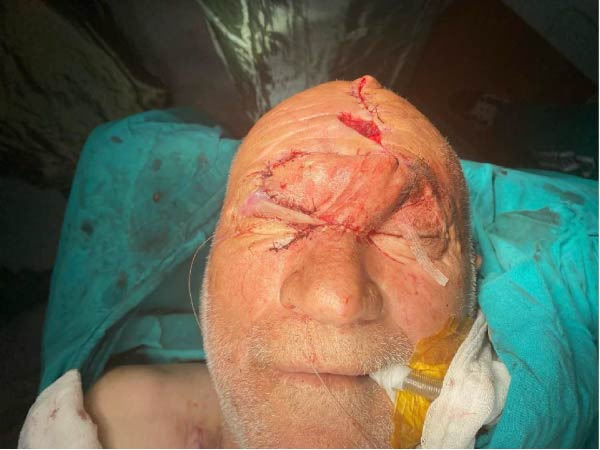
After reconstruction.

Intraoperative examination demonstrated tumor infiltration of the lacrimal sac. A 2 cm × 1 cm bone window was created at the junction of the nasal bone and the nasomaxillary suture to allow visualization and ligation of the nasolacrimal duct, which was subsequently included in the surgical specimen. Excision proceeded along the upper eyelid, superficial to the orbital septum. Frozen section analysis demonstrated tumor extension beyond the brow border and into the frontal region within the subperiosteal plane, requiring further widening of the surgical margins. (Figure 3 and 4) Frozen section analysis was performed circumferentially, and additional margins were excised until tumor‐free margins were confirmed. After achieving complete tumor excision with negative frozen section results, the nasolacrimal sac was reconstructed using a dacryocystorhinostomy (DSR) tube, and the bone window was reattached with PDS sutures. To reconstruct the excised medial canthal tendon and prevent telecanthus deformity, remnants of the medial canthal tendon and residual conjunctiva were suspended to the nasal bone. The medial and lateral parts of the conjunctiva were sutured to cover the globe. The superior tarsus and levator palpebrae superioris muscle were preserved. A contralateral forehead flap was designed to reconstruct a 9 cm × 5 cm defect encompassing the upper and lower eyelids, palpebral conjunctiva, frontal region, eyebrow, nasal radix, and nasal dorsum (Figure 5 and 6).

**Figure 7:**
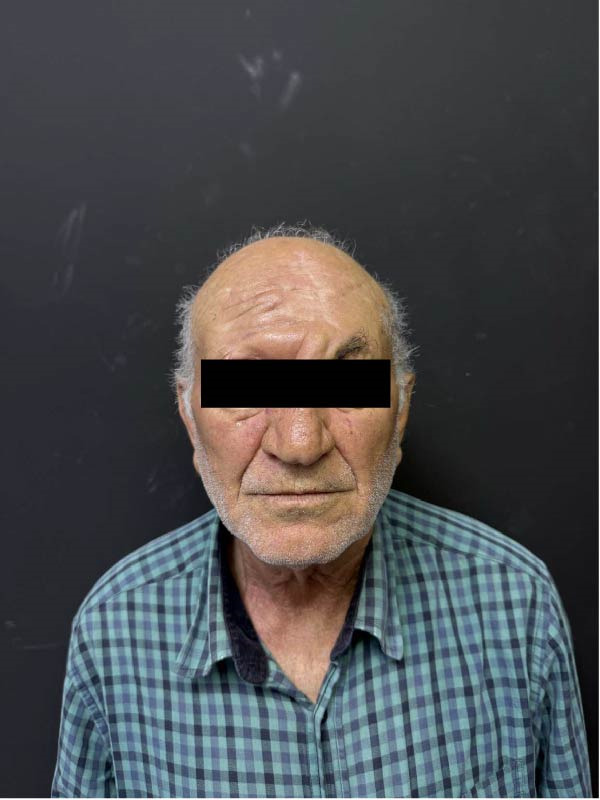
Postoperative appearance at 9‐month follow‐up.

At the 9‐month follow‐up, no clinical evidence of local recurrence was observed. The patient did not report epiphora, and no aesthetic revision was requested. Overall, the patient expressed satisfaction with the aesthetic outcome (Figure 7).

## 3. Discussion

In the literature, the morpheaform subtype accounts for less than 10% of all BCCs [4]. In particular, people over 60 are frequently affected [5]. Based on German registry data, the age‐standardized incidence in men and women is 122.4 and 152.2 per 100,000 (European standard), respectively [6].

While 80% of BCCs are localized in the head and neck, 20% are localized in the eyelids [7]. In eyelid BCC, 50% of lesions are found on the lower lid, 30% on the medial canthus, 15% on the upper lid, and 5% on the lateral canthus [8].

Anatomic location in the H‐zone and periorbital area, aggressive BCC subtypes such as micronodular and morpheaform have been known to be the risk factor for BCC recurrence [9].

There are a number of reported cases of BCC invasion of the lacrimal caruncle, lacrimal gland, or nasolacrimal duct. There are also several reported cases of primary BCC arising in the lacrimal system. However, BCC invasion of the lacrimal canaliculus or lacrimal sac is rare, with only six previously reported cases [10].

Considering all these, thorough imaging and ophthalmologic evaluation are essential prior to tumor resection in this region.

Morpheaform type BCC may cause difficulty in establishing adequate surgical margins because it progresses aggressively under the skin. Therefore, it is very important to perform Mohs microsurgery or frozen section analysis if possible, to inform the patient about the size and consequences of the defect before surgery, and to prepare the team for large defects and reconstruction of these possible defects.

In the literature, full‐thickness skin grafts for patients with only skin defects, local flaps from the glabellar‐nasal region, forehead flaps, and fascia grafts for larger defects, including lacrimal system, medial canthal tendon, nasal‐maxillary bone, etc., have been used in the reconstruction of defects after BCC excisions in the medial canthal region. More extensive and complex reconstruction options, such as anterolateral thigh flap, radial forearm free flap, rectus abdominis musculocutaneous flap, and temporal flap, are applied in cases of orbital exenteration with bulbar involvement [11–14].

As described above, frozen section‐guided excision of a patient with locally advanced morpheaform BCC in the medial canthal region resulted in a large defect involving the lacrimal duct and sac, upper and lower eyelid, medial canthal tendon, part of the medial conjunctiva, and frontal region.

In this case, considering the infiltrative structure of the lesion, resection was performed in the subperiosteal plane. The nasal bone was elevated to reach the lacrimal sac and then replaced. A DSR tube was placed for nasolacrimal canal reconstruction, and a portion of the conjunctiva and eyelid defect was partially reduced by advancement. Then, a contralateral forehead flap was designed for the reconstruction of the remaining large defect, and the defect was reconstructed.

In such large defects, the forehead flap is a reliable reconstruction option considering its reliable blood supply, adequate soft tissue coverage, the ability to provide tissue in different thicknesses depending on the defect area, and the fact that the donor area can be largely closed with primary repair, especially in elderly patients.

This report is limited by its nature as a single case, and therefore the findings cannot be generalized. Nevertheless, given the rarity of lacrimal sac invasion by morpheaform BCC, this case provides valuable clinical insight into diagnosis, surgical planning, and reconstruction of aggressive periocular tumors.

## Funding

This research received no specific grant from any funding agency in the public, commercial, or not‐for‐profit sectors.

## Ethics Statement

Ethics committee approval was waived as this study represents a single case report, in accordance with institutional policy.

## Consent

Written informed consent was obtained from the patient for publication of this case report and accompanying clinical images.

## Conflicts of Interest

The authors declare no conflicts of interest.

## Data Availability

The data that support the findings of this study are available upon request from the corresponding author. The data are not publicly available due to privacy or ethical restrictions.
